# Movement of *Salmonella* serovar Typhimurium and *E. coli* O157:H7 to Ripe Tomato Fruit Following Various Routes of Contamination

**DOI:** 10.3390/microorganisms3040809

**Published:** 2015-11-05

**Authors:** Amanda J. Deering, Dan R. Jack, Robert E. Pruitt, Lisa J. Mauer

**Affiliations:** 1 Department of Food Science, Purdue University, 745 Agriculture Mall Drive, West Lafayette, IN 47907, USA; E-Mails: DJack@glgroup.com (D.R.J.); mauer@purdue.edu (L.J.M.); 2 Department of Botany and Plant Pathology, Purdue University, 915 W. State St., West Lafayette, IN 47907, USA; E-Mail: pruittr@purdue.edu

**Keywords:** tomato, produce contamination, pathogen internalization, produce safety, pathogen contamination, foodborne illness

## Abstract

*Salmonella* serovars have been associated with the majority of foodborne illness outbreaks involving tomatoes, and *E. coli* O157:H7 has caused outbreaks involving other fresh produce. Contamination by both pathogens has been thought to originate from all points of the growing and distribution process. To determine if *Salmonella* serovar Typhimurium and *E. coli* O157:H7 could move to the mature tomato fruit of different tomato cultivars following contamination, three different contamination scenarios (seed, leaf, and soil) were examined. Following contamination, each cultivar appeared to respond differently to the presence of the pathogens, with most producing few fruit and having overall poor health. The Micro-Tom cultivar, however, produced relatively more fruit and *E. coli* O157:H7 was detected in the ripe tomatoes for both the seed- and leaf- contaminated plants, but not following soil contamination. The Roma cultivar produced fewer fruit, but was the only cultivar in which *E. coli* O157:H7 was detected via all three routes of contamination. Only two of the five cultivars produced tomatoes following seed-, leaf-, and soil- contamination with *Salmonella* Typhimurium, and no *Salmonella* was found in any of the tomatoes. Together these results show that different tomato cultivars respond differently to the presence of a human pathogen, and for *E. coli* O157:H7, in particular, tomato plants that are either contaminated as seeds or have a natural opening or a wound, that allows bacteria to enter the leaves can result in plants that have the potential to produce tomatoes that harbor internalized pathogenic bacteria.

## 1. Introduction

In recent years, there have been many foodborne illness outbreaks associated with fresh fruits and vegetables. Between 1996 and 2008, 82 outbreaks were associated with the consumption of fresh produce. Among these outbreaks, 17% were linked to tomatoes and caused over 1900 illnesses and three deaths [1]. Although *Shigella* spp. have been implicated in some of the tomato-related outbreaks [2], the majority of the outbreaks linked to tomatoes have been associated with *Salmonella* serotypes [3]. Several of the outbreaks have been linked to on-farm contamination, while in others the bacterium was thought to have been introduced later from the packing facilities or during food preparation [4,5]. This shows that the risk for *Salmonella* spp. contamination is likely present at every point in the growing and distribution process, which increases the probability of causing illness to consumers.

A number of *Salmonella* serovars, including Javiana, Montevideo, and Newport, have been linked to tomato-associated outbreaks [3]. Guo *et al.* (2001) examined how well various serovars of *Salmonella* could colonize growing tomato plants [6]. The serovars were introduced onto the flowers of the tomato plants, and the fruit produced then sampled for the presence of *Salmonella*. Serovar Poona was found associated with the external portions of the tomato, while serovar Montevideo was found associated with both the outside and internalized within the tomato fruit. Other studies have also shown that *Salmonella* introduced directly onto the flowers of plants could be detected both on the surface and internalized within the fruit produced from each flower [7,8,9]. These data suggest that *Salmonella* has the ability to become established on or within a tomato fruit following contamination after flowering. In addition, contamination can be serovar specific, with *S*. Montevideo being able to survive and persist on and within the tomato fruit better compared to other serovars suggesting adaptation to the tomato environment [9].

*E. coli* O157:H7 can be tolerant and adaptable to acidic environmental conditions, which allows the bacterium to survive better in acidic foods and beverages in comparison to other bacteria commonly associated with food-related outbreaks [10]. A number of studies have determined that *E. coli* O157:H7 is able to survive in various acidic foods [11], and that survival is prolonged upon storage of the food at refrigeration temperatures as compared to room temperature. Eribo and Ashenafi (2003) examined the survival of *E. coli* O157:H7 in fresh tomatoes and various tomato products and determined that the bacterium can survive for 10 days in fresh whole tomatoes and for 16 days in processed whole tomatoes under refrigerated storage conditions [12]. Given these results, as well as the number of outbreaks that have occurred involving *E. coli* O157:H7 with other fresh produce items [13,14,15], examining possible interactions (such as survival, internalization within the plant, and movement to the fruit) between this bacterium and tomato plants/fruits is warranted.

Various routes of pathogen contamination on tomato plants have been tested including that by aerosols formed by rain, leaf tissue inoculated with a surfactant, and even the effect of soil management [16,17,18]. In this study, the movement of *Salmonella* serovar Typhimurium and *E. coli* O157:H7 from the plant into the ripe tomato fruit was examined via three different modes of contamination: contamination of the intact seed, leaf infiltration to add bacteria to the inner portions of the leaf, and contamination of the soil surrounding the plant. Each of these methods involved using liquid media as the contamination source to mimic conditions where the plant is exposed to contaminated irrigation water. Polymerase Chain Reaction (PCR) based techniques were then used to detect the presence of the bacteria from the sampled ripe tomatoes. Different cultivars of tomato were also used to examine if any cultivar specificity was present between the bacteria and the different modes of contamination. This study will provide information as to how well *E. coli* O157:H7 and *Salmonella* Typhimurium are able to persist and move within tomato plants. In addition, this information will provide insight into understanding if the bacteria are able to move from the plant and ultimately be internalized within the ripe fruit, which may contribute to the observed increase in outbreaks associated with tomatoes.

## 2. Experimental Section

### 2.1. Bacteria

In order to track inoculated pathogens in the tomato plants, strains expressing GFP (green fluorescent protein) were used. *Escherichia coli* O157:H7 B6-914 GFP-91 (further referred to as *E. coli* O157:H7-GFP) was used in this study. The strain was constructed by transforming *E. coli* O157:H7 B6-914 with the GFP plasmid pGFP (cDNA vector, Clontech, Mountain View, CA, USA) containing the ampicillin resistance gene for selection. This strain does not produce Shiga-like toxins Stx1 and Stx2 and was chosen for laboratory safety advantages. Differences in growth kinetics between the strain used and the parental strain have not been observed [19]. In addition, the lack of the toxin genes *stx1* and *stx2* has have been shown to not change the *E. coli* O157:H7 growth characteristics [20]. Bacteria were stored at −80°C as 7% dimethyl sulfoxide (DMSO) freezer stocks and cultured in Luria–Bertani (LB) broth supplemented with ampicillin (100 μg/mL; LB + AMP) at 37 °C.

For the construction of *Salmonella* Typhimurium-GFP, pGFP plasmid DNA was isolated from a 1.5 mL culture of *Escherichia coli* O157:H7 B6-914 GFP-91 grown in Luria-Bertani medium containing 100 μg/mL ampicillin (LB + AMP) using a QIAprep Spin Miniprep kit according to the manufacturer’s directions. Approximately 1 ng of this DNA was added to 50 μL of electrocompetent *Salmonella enterica* serovar Typhimurium prepared according to the recommendation of the manufacturer of the electroporation apparatus (Bio-Rad, Hercules, CA, USA) and then pulsed at 2.5 kV in a 0.2 cm electroporation cuvette. 1 mL of SOC medium (2% Bacto™ tryptone (Becton, Dickinson and Company, Sparks, MD, USA), 0.5% Bacto™ yeast extract, 10 mM NaCl, 2.5 mM KCl, 10 mM MgCl_2_, 10 mM MgSO_4_, 20 mM glucose) was added and the cells and allowed to recover for 1 h at 37 °C before being plated on LB + AMP plates and incubated overnight at 37 °C. Colonies expressing GFP were observed using long-wave UV light, picked into fresh 1.5 mL LB + AMP cultures, and incubated overnight at 37 °C. DNA was extracted from the cells by pelleting a 1 mL aliquot of the overnight culture and resuspending the pellet in 500 μL SM Buffer (0.85% NaCl, 1 mM MgSO_4_). Five hundred μL of 2X TZ solution (cell lysis solution; 4% Triton-X, 5 mg/mL Sodium Azide in 0.1 M Tris-HCl pH 8.0) was then added and the tubes boiled for 10 min. The samples were centrifuged at 10,000 g for 5 min and the supernatant containing the DNA removed. PCR was then performed using primers to *invA* to verify that the colonies were *Salmonella enterica* [21,22].

### 2.2. Tomato Varieties

Tomato seeds were obtained from Tomato Growers Supply Company (Ft. Meyers, FL, USA). The five dwarf tomato varieties used for the *E. coli* O157:H7 portion of this study were: Micro-Tom, Tommy Toe, Elfin, Sweet Quartz, and Red Currant. Roma was the only non-dwarf variety used. The *Salmonella* Typhimurium portion of the study used the same cultivars with the exception of Roma. Descriptions of the cultivar traits are available from the supplier.

### 2.3. Inoculation of Plants and Growing Conditions

Overnight cultures of *E. coli* O157:H7-GFP and *Salmonella* Typhimurium-GFP were grown in LB + AMP at 37 °C with shaking. The culture was washed three times to remove the growth media; cells were centrifuged for 6 min at 3000 rpm, the supernatant was removed, and the resulting pellet was resuspended in 0.1 M Phosphate Buffer pH 7.0 by vortexing. The bacteria were then enumerated by plating on LB + AMP plates and incubating overnight at 37 °C. Five tomato plants per cultivar were contaminated by three different routes in separate experiments with either *E. coli* O157:H7-GFP or *Salmonella* Typhimurium-GFP, as described below. *E. coli* and *Salmonella* inoculation experiments were not run concurrently as there was limited Biosafety level 2 space in the greenhouse. Plants for each experiment were all arranged on the same greenhouse bench space but control plants were placed apart from experimental plants to ensure no contact between the two. The GFP containing plasmid has been shown to be stable on non-selective bacterial growth media [23] but could be less stable when growing on plants. Hence, the results presented here may underestimate the level of persistence of these human pathogenic bacteria in the plant.

#### 2.3.1. Treatment for Seed-Contaminated Plants

Tomato seeds were soaked in 10^7^ CFU/mL of the washed *E. coli* O157:H7-GFP or 10^5^ CFU/mL of the washed *Salmonella* Typhimurium-GFP solution for 30 min. The seeds were then dried on filter paper at room temperature for 1 h prior to placement into pots containing SunGro^®^ Sunshine^®^ Mix #4 (Sun Gro Horticulture, Agawam, MA, USA) soil fertilized with Osmocote^®^ (The Scotts Company, Marysville, OH, USA). The plants were grown under greenhouse conditions maintained at an average temperature of 28 °C with a 16/8 light-dark cycle.

#### 2.3.2. Treatment for Soil-Contaminated Plants

Tomato seeds were planted into pots containing SunGro^®^ Sunshine^®^ Mix #4 soil fertilized with Osmocote^®^ and allowed to grow under specified greenhouse conditions. The plants were soil contaminated by adding 5 mL of the washed culture (the initial concentration of *E. coli* O157:H7-GFP was 10^7^ CFU/mL; the initial concentration of *Salmonella* Typhimurium-GFP was 10^5^ CFU/mL) around the base of the plant. The plants were soil contaminated at 44 days after planting for the *E. coli* O157:H7-GFP treated plants and at 32 days after planting for the *Salmonella* Typhimurium *r*-GFP treated plants. The day of contamination was based on the growth rate of the plants (flowering but no fruit production).

#### 2.3.3. Treatment for Leaf-Contaminated Plants

Tomato seeds were planted into pots containing SunGro^®^ Sunshine^®^ Mix #4 soil fertilized with Osmocote^®^ and allowed to grow under specified greenhouse conditions. At 44 days after planting for the *E. coli* O157:H7-GFP contaminated plants and 32 days after planting for the *Salmonella* Typhimurium*r*-GFP contaminated plants, the underside of each leaflet (typically a total of five leaflets per leaf) from one randomly chosen leaf on each plant was infiltrated with ~200 μL of a washed culture of each bacterium (the initial concentration of *E. coli* O157:H7-GFP was 10^7^ CFU/mL; the initial concentration of *Salmonella* Typhimurium-GFP was 10^5^ CFU/mL) using a sterile syringe without a needle. The leaf was marked for later identification.

#### 2.3.4. Treatment for Control Plants

The control plants used in the experiments were grown as described for each of the three modes of contamination. However, instead of introducing *E. coli* O157:H7-GFP or *Salmonella* Typhimurium-GFP as described for the other experimental treatments, the seeds or plants were treated with 0.1 M Phosphate Buffer pH 7.0 only. The control plants were grown under the same greenhouse conditions as the experimental plants, but were grown in separate trays and did not come in contact with the plants contaminated with the GFP bacteria.

### 2.4. Sampling of Ripe Tomato Fruit

For plants treated with *E. coli* O157:H7-GFP, the soil- and leaf- contaminated plants were sampled starting at 35 days following contamination, and the seed-contaminated plants were sampled starting at 79 days following seed contamination. For the plants treated with *Salmonella* Typhimurium-GFP, sampling began at 44 days for the soil- and leaf- contaminated plants and at 76 days for the seed-contaminated plants. Proper care was exercised during fruit sampling so as not to contact other parts of the plant and only ripe, red tomatoes were picked for all samples taken. Every tomato present on each cultivar was counted, harvested, and ground in 10 mL of 0.1 M phosphate buffer using a mortar and pestle. The samples were incubated overnight at 37 °C. The samples were then plated (100 μL) on LB + AMP plates and incubated overnight at 37 °C. To obtain the liquid cultures that were used for the whole cell PCR, a representative mixture of colonies from the plates were picked into 3 mL of LB + AMP broth and incubated overnight at 37 °C with shaking.

### 2.5. PCR for the Detection of GFP

PCR-based methods were used for detection due to the variability of GFP expression when the bacteria are recovered from plants [24]. Amplification reactions were performed in a final volume of 20 μL containing 2 μL of the overnight culture (whole cells), 200 μmol dNTPs (Promega, Madison, WI, USA), PCR Buffer (final concentration: 50 mM KCl, 10 mM Tris pH 9.0, 0.1% Triton X-100, 2 mM MgCl_2_), 0.5 units of DNA-Free Taq DNA polymerase (Bioron, Ludwigshafen, Germany) and 5 pmol each of the forward and reverse primers. The following primer set was used: GFP FORWARD: 5′-GCCCGAAGGTTATGTACAGG-3′, GFP REVERSE: 5′-AAAGGGCAGATTGTGTGGAC-3′ (Integrated DNA Technologies, Inc., Coralville, IA, USA). PCR amplification was performed using a PTC-100™ programmable thermal controller (MJ Research, Inc., Waltham, MA, USA) with the temperature cycling as follows: 95 °C for 2 min, followed by 35 cycles of denaturation at 94 °C for 30 s, annealing at 58 °C for 25 s, and extension at 68 °C for 1 min with a final extension at 68 °C for 10 min. The PCR products were size-separated by Tris-Borate-Ethylenediaminetetraacetic acid (EDTA)-buffered agarose gel electrophoresis. Agarose (2%) gels were run for 1.5 h at 120 V in 1X TBE running buffer (89 mM Tris-Base, 89 mM Boric Acid, 2 mM EDTA pH 8.9).

### 2.6. Confirmation of E. coli O157:H7-GFP

Representative cultures that tested positive for GFP by PCR analysis were also plated on CT-SMAC (cefixime tellurite sorbitol MacConkey agar) plates to confirm the presence of *E. coli* O157:H7 in the sample. The cultures (100 μL) were plated on Sorbitol MacConkey agar (Difco™, BD Diagnostics, Sparks, MD, USA) supplemented with Cefixime (0.05 mg/L) and Potassium Tellurite (2.5 mg/L) and incubated overnight at 37 °C. The plates were examined for the presence of colorless colonies indicating the presence of *E. coli* O157:H7.

### 2.7. Persistence of E. coli O157:H7-GFP *and* Salmonella Typhimurium-GFP

The growth and persistence of *E. coli* O157:H7 and *Salmonella* associated with tomato plants was also of interest and was determined by contaminating seeds, allowing them to germinate, and then analyzing the plant tissue. Washed cultures of *E. coli* O157:H7-GFP and *Salmonella* Typhimurium-GFP were prepared as described above. In separate experiments, Micro-Tom tomato seeds were soaked for 30 min in a 10^8^ CFU/mL culture of *E. coli* O157:H7-GFP and a 10^6^ CFU/mL culture of *Salmonella* Typhimurium-GFP at room temperature with rotation. The seeds were removed and allowed to dry on filter paper for one hour. For initial contamination data, five seeds were ground using a mortar and pestle in 10 mL of 0.1 M Phosphate Buffer pH 7.0, plated on LB + AMP plates, and incubated overnight at 37 °C. The remaining seeds were individually placed into sterile 25 mm × 150 mm test tubes containing 25 mL of sterile 0.8% soft-top agar. The tubes were sealed using Parafilm M^®^ (Bemis Flexible Packaging, Neenah, WI, USA) and incubated in a growth chamber at 25 °C with 50% relative humidity and a 16/8 light cycle. At 3, 6, 9, and 12 days after contamination, five plants were removed and ground in 10 mL of 0.1 M Phosphate Buffer pH 7.0 using a mortar and pestle. The number of bacteria from each plant was enumerated by plating on LB + AMP plates, incubating overnight at 37 °C and scoring the resulting colonies for the presence of GFP under UV light. The average CFU/plant was then determined for each sample time.

### 2.8. Statistical Analysis

The data were analyzed using ANOVA models to evaluate if differences were present between cultivars and contamination routes. Individual differences were tested using Tukey’s multiple means comparison procedure. All statistical analysis procedures were conducted using PC SAS 9.3 (SAS Institute, Inc., Cary, NC, USA) with α = 0.05.

## 3. Results and Discussion

### 3.1. Contaminating Tomato Plants with E. coli O157:H7*-GFP* and Salmonella Typhimurium-GFP Affected Plant Health and Tomato Production

**Figure 1:**
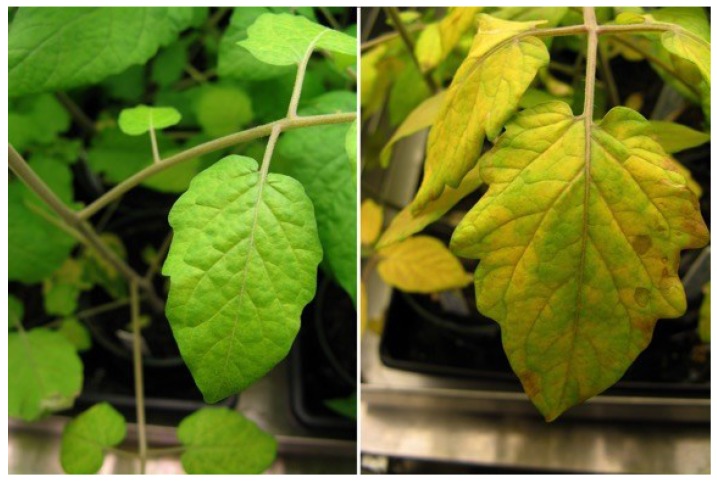
Example of a control tomato plant (**left**) compared to a contaminated tomato plant (**right**) grown under the same conditions for the same time period.

**Figure 2:**
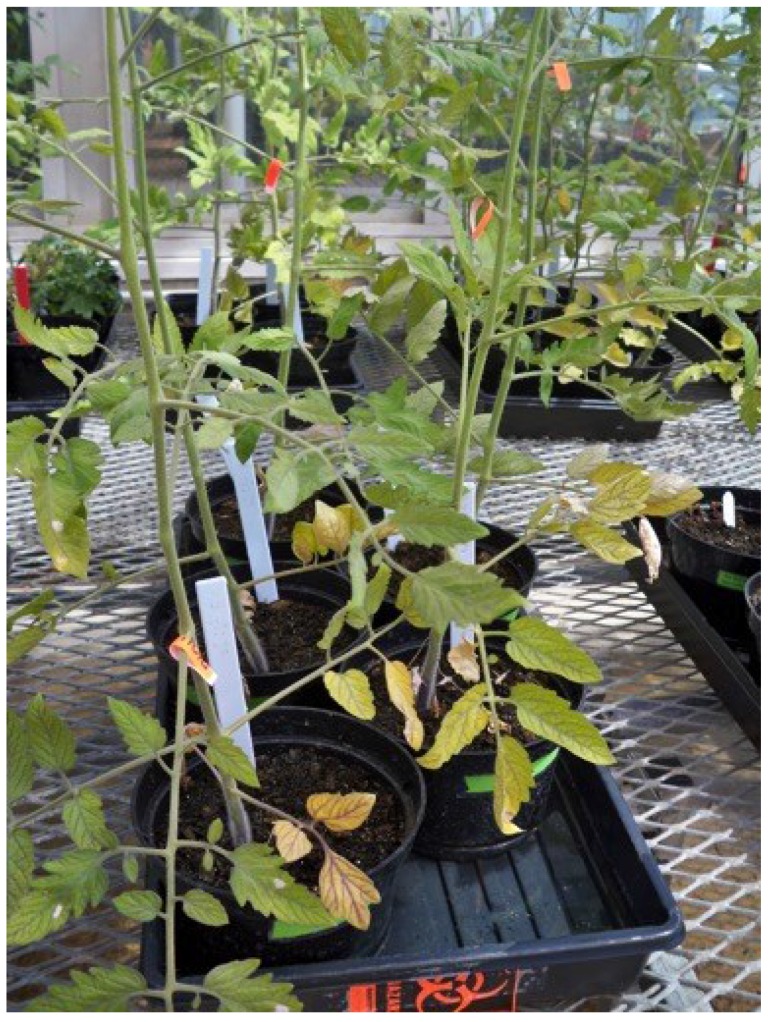
Red Currant tomato plants that had been leaf inoculated with *E. coli* O157:H7-GFP.

An overarching observation throughout this study was that the contamination of tomato plants by *E. coli* O157:H7-GFP affected the health of four of the six tomato cultivars used and *Salmonella* Typhimurium-GFP affected the health of four of the five cultivars used (Figure 1). Almost all of the plants that had been treated with the bacteria through the three routes of contamination, with the exception of the Micro-Tom plants, had many yellow leaves with small necrotic lesions present, and were in poor overall health. The Micro-Tom and Roma were the only cultivars that grew relatively well following contamination by any route, produced many fruit, and seemed relatively healthy throughout the course of the experiment (Figure 2). The other tomato cultivars did not produce many fruit, if any, following contamination, which could likely be attributed to the overall poor health of the plants. Other studies involving pathogen inoculation on produce have reported poor plant health with various symptoms such as stunted growth or diminished root formation [25].

**Figure 3:**
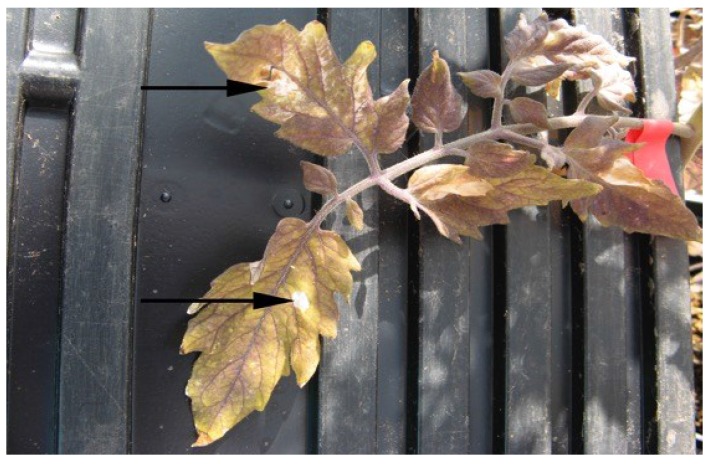
Tomato leaf from a plant that had been leaf inoculated with *Salmonella* Typhimurium-GFP. The arrow marks a necrotic lesion on the plant that resulted from the point of inoculation on the leaflet.

A hypersensitive response was observed with all of the tomato cultivars following leaf infiltration with the bacterial culture. The hypersensitive response is characterized by a rapid death of the cells (necrosis) in the area locally surrounding a site of infection and is a mechanism used to prevent the spread of the microbial pathogen throughout the plant [26]. The necrotic lesions were observed on each of the leaflets within one week following leaf infiltration of the bacteria (Figure 2). After approximately two weeks, the entire leaf started to become necrotic, and at the time the tomatoes were sampled (35 days following inoculation for *E. coli* O157:H7-GFP and 44 days for following inoculation for *Salmonella* Typhimurium-GFP) the infiltrated leaves on all plants were completely dead and no longer attached to the plant (Figure 3).

### 3.2. Effects of Routes of Contamination by E. coli O157:H7*-GFP*

**Figure 4:**
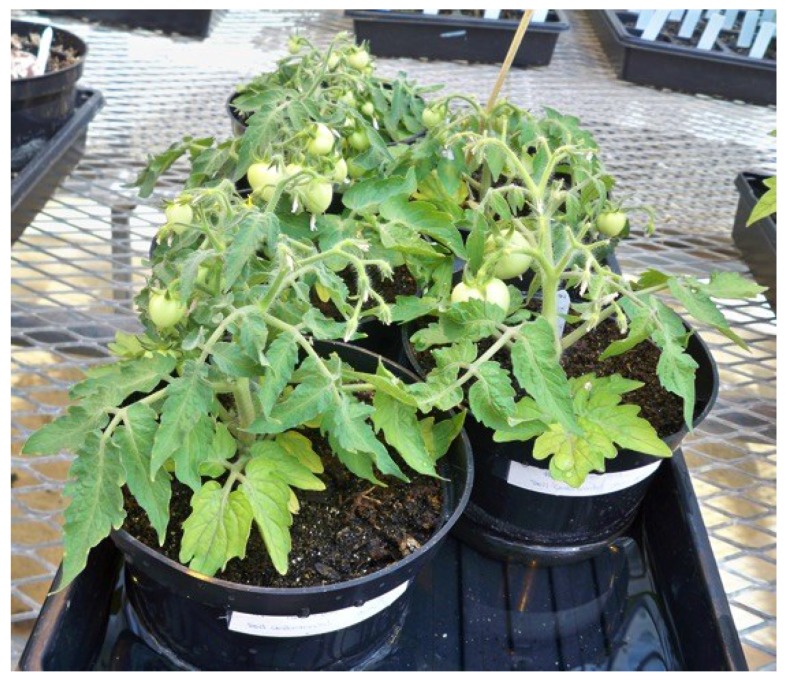
Micro-Tom plants that had been seed-contaminated with *E. coli* O157:H7-GFP.

**Figure 5:**
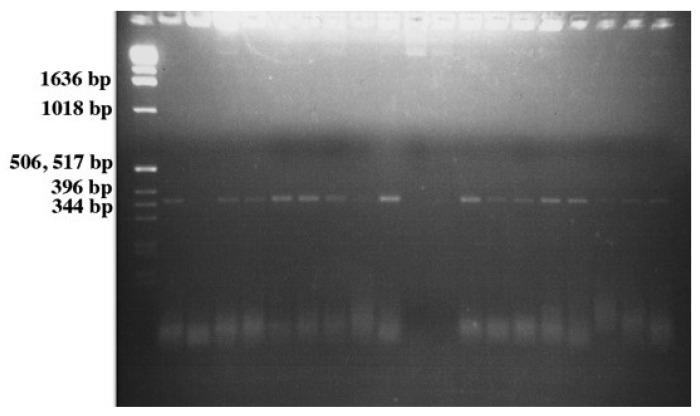
Representative agarose gel showing the PCR products using the GFP specific primers from cells recovered from the sampled tomatoes. Product size = 359 base pairs (bp).

A total of 242 tomatoes were produced by and sampled from plants that were seed, soil, and leaf contaminated with *E. coli* O157:H7 (Table 1). Significant differences (α = 0.05) were found in the numbers of tomatoes produced by different cultivars following different routes of contamination. Tomatoes were present on all of the cultivars examined, however, the number of fruit set was very low due to poor plant health for all of the cultivars except Micro-Tom, and to a lesser extent, Roma (Figure 4). A total of 72 tomatoes were produced by and sampled from the five Micro-Tom plants that were seed contaminated, with 54% of these tomatoes being positive for GFP indicating the presence of *E. coli* O157:H7 in the mature fruit. A representative gel showing the GFP PCR products (product size = 359 base pairs) is shown in Figure 5. In addition, 49 tomatoes were sampled from Micro-Tom plants that were leaf inoculated with the bacterium, and 39% were positive for GFP. However, 57 Micro-Tom tomatoes were sampled from plants that were soil contaminated and none of the tomatoes tested positive for GFP. Significant differences (α = 0.05) in the percentages of contaminated fruit following different routes of contamination were found, suggesting that the mode of contamination is important in determining if the bacteria can move into the mature tomato fruit of a contaminated plant. This may be explained by the fact that at the point of soil contamination the plant had mature natural barriers, such as the Casparian strip present in the root system that prevents various unwanted organisms and molecules from entering the plant [27]. This natural barrier, and others such as the waxy cuticle covering the plant, are not mature when seeds are contaminated and therefore would have little influence on the ability of the bacteria to be internalized within the plant. In addition, the bacteria do not have to evade these natural barriers during leaf contamination as the bacteria are put directly into the inner mesophyll portions of the leaf during inoculation. This may explain why the *E. coli* O157:H7-GFP were observed in the tomato fruit of the seed and leaf contaminated Micro-Tom plants, but not found in the tomatoes from the soil contaminated plants. The Roma cultivar was the only cultivar to show that *E. coli* O157:H7-GFP was able to move to the fruit via all three modes of contamination. The Roma plants produced a total of 51 tomatoes. Of the 12 tomatoes produced by soil contaminated plants, 50% were positive for *E. coli* O157:H7-GFP. Seed contaminated Roma plants produced the most fruit (25 tomatoes) of the three modes for this particular cultivar, of which 40% tested positive for GFP. Leaf contaminated Roma plants produced 14 tomatoes with a 21.4% GFP positive rate. This demonstrates that there are certain cultivars, of which Roma is one, that could allow successful fruit contamination via multiple routes. Such cultivars should be similarly tested in a production setting since they might be more likely to harbor any pathogens introduced via the environment.

The other tomato cultivars produced fruit following contamination with *E. coli* O157:H7-GFP, however, not with every mode of contamination (seed, soil, and leaf), as documented in Table 1. Red Currant and Sweet Quartz only produced fruit in the seed contaminated plants. The seed contaminated Red Currant plants produced five fruit with 60% testing positive for GFP. The seed contaminated Sweet Quartz plants produced only three tomatoes; however, all (100%) were positive for GFP. The leaf contaminated Elfin plants were the only group that produced fruit for that cultivar. Only two tomatoes were produced and neither (0%) tested positive for GFP. For the Tommy Toe plants, only the soil contaminated plants produced fruit (three total), and of them 67% tested positive for GFP. This was the only cultivar, other than Roma, that produced fruit in the soil contaminated plants in which the GFP could be detected in the mature fruit (57 tomatoes were sampled from the soil contaminated Micro-Tom plants and none were positive for GFP). This suggests that there may be differences between the cultivars that allow the bacteria to move to the mature fruit following soil contamination in the Tommy Toe and Roma cultivars, but not in others (Table 1). Previous studies have demonstrated there are differences in how well various serotypes of *Salmonella* spp. are able to be internalized within a tomato fruit following flower inoculation [6,28]. However, few studies have addressed if there is plant-cultivar specificity present between the plants tested that would allow some cultivars to harbor internalized bacteria more effectively than others. Barak *et al*. (2011) reported that type I trichomes of tomato plants were preferred colonization sites for *S. enterica* and Cevallos-Cevallos *et al*. (2012) reported improved *Salmonella* survival on high trichome density leaves [29,30]. Such differences in trichome density, surface topography, maturation periods or other varying tomato cultivar features could affect pathogen colonization and potential subsequent internalization and translocation capabilities. Although the mechanism of how internalization may occur remains unclear, finding bacteria internalized in or on the surface of tomatoes of two cultivars that were soil contaminated, and not from the other four tested, suggests that there may be some cultivar specificity that allows the bacteria to more easily colonize the Tommy Toe and Roma varieties than others. Tomato cultivars have previously been shown to exhibit influence on pathogen survival [31].

**Table 1:** Total number of tomatoes produced by and sampled from five plants of each tomato cultivar that had been contaminated (seed, soil, or leaf) with *E. coli* O157:H7-GFP, and the percent positive for GFP following PCR analysis.

Cultivar	Contamination Type	Total Tomatoes Produced (and Sampled)	% GFP Positive
Micro-Tom	Seed	72	54.2%
	Soil	57	0%
	Leaf	49	38.8%
Tommy Toe	Seed	0	-
	Soil	3	66.7%
	Leaf	0	-
Red Currant	Seed	5	60.0%
	Soil	0	-
	Leaf	0	-
Sweet Quartz	Seed	3	100%
	Soil	0	-
	Leaf	0	-
Roma	Seed	25	40%
	Soil	12	50%
	Leaf	14	21.4%
Elfin	Seed	0	-
	Soil	0	-
	Leaf	2	0%
**Total sampled**	**-**	**242**	**35.1%**

**Figure 6:**
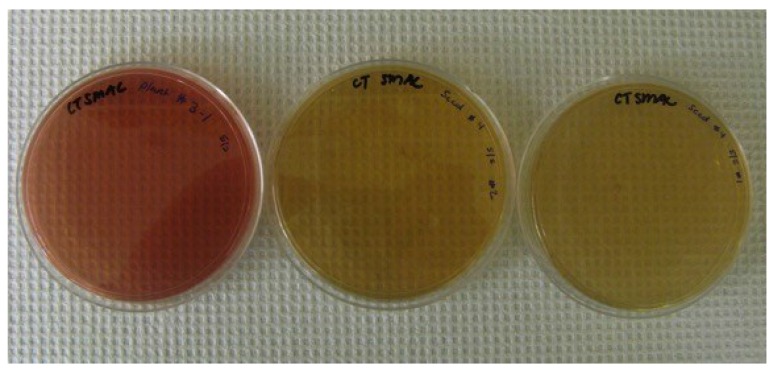
CT-SMAC plates from *E. coli* O157:H7-GFP samples recovered from tomatoes that had been seed contaminated with the bacterium (two plates on the right) and were positive for GFP following PCR. *E. coli* O157:H7 does not ferment sorbitol and appears as white colonies on CT-SMAC plates. As a negative control, cultures were plated that were not positive for GFP. The plate on the left is representative of the plates obtained with colonies all appearing red in color (can ferment sorbitol) indicating that they are not *E. coli* O157:H7.

Cultures grown from the tomatoes that tested positive for GFP were also plated on CT-SMAC plates to further confirm the presence of *E. coli* O157:H7-GFP in the tomato fruit. Two plates that were plated from a positive GFP sample from the PCR analysis are shown in Figure 6. Many colonies were present on the plate and all were colorless (turning the overall color of the plate to near colorless), indicating these samples do not ferment sorbitol and were positive for *E. coli* O157:H7. As a control for comparison, another culture grown from a tomato that did not test positive for GFP following PCR analysis was also plated on CT-SMAC and all the colonies present were red (giving the plate an overall red color), indicating that these bacteria can ferment sorbitol and that *E. coli* O157:H7 was not present in the sample.

In the control plants (plants treated with phosphate buffer only), nine tomatoes were sampled from three different plants for each treatment (seed, soil, and leaf). A second set of control experiments were performed for all three routes of contamination for Roma and Micro-Tom where 20 tomatoes from each route of contamination were sampled. None of these 120 tomatoes from the control plants were positive for GFP. This provides further support that the GFP observed in the tomatoes sampled from the contaminated plants was from the bacteria that were able to move from the plant into the mature tomato fruit.

### 3.3. Effects of Routes of Contamination by Salmonella Typhimurium-GFP

Very different results were obtained from the tomato plants that were contaminated with *Salmonella* Typhimurium-GFP compared to those contaminated with *E. coli* O157:H7-GFP. Only the Micro-Tom and Elfin cultivars produced tomatoes for any of the modes of *Salmonella* Typhimurium-GFP contamination (seed, soil, leaf). The same poor overall health of the plants was observed with the *Salmonella* Typhimurium-GFP contaminated plants as was observed with the plants contaminated with *E. coli* O157:H7, which may explain the low number of tomatoes produced by all of the cultivars. A combined total of 121 tomatoes were produced by and sampled from the Micro-Tom and Elfin plants, and GFP was not detected in any of the tomatoes sampled (0%). However, bands were observed in the positive control samples indicating the primers and PCR conditions were correct. A total of nine tomatoes were also sampled from three different control plants of each cultivar for each of the *Salmonella* Typhimurium-GFP treatments (seed, soil, and leaf). Again, GFP was not detected in any of these tomatoes. Together these data suggest that *Salmonella* Typhimurium-GFP is not able to move to the mature tomato fruit following contamination of the plant via the seed, leaf, or soil and/or that *Salmonella* Typhimurium-GFP is not able to colonize the five cultivars tested in this study. Zheng *et al.* (2013) conducted soil, leaf and blossom contamination of Micro-Tom cultivar tomato plants with a five serovar cocktail of *Salmonella*. While four of the serovars were found to effectively colonize niche areas of the plant, *Salmonella Typhimurium*, specifically, was not recovered in the fruit and was found to have a poor survival rate which corroborates the data in this study [28]. Although *Salmonella* Typhimurium has not been associated with as many outbreaks as other serovars reported [32], it has been shown to grow on tomato fruit as well as other outbreak strains (s.v. Newport, Braenderup, Javiana, and Montevideo) that were tested [33]. This demonstrates the ability of *Salmonella* Typhimurium to grow on tomatoes; however, the tomato cultivar used in the previous study was Campari. Additionally, Gu *et al*. (2013) showed the ability of *Salmonella* Typhimurium to enter hydathodes on tomato leaves and make their way into the vascular system of the plant. The cultivars used in the study, however, also differed from the ones used here [31]. This suggests that there is a cultivar specificity present where some cultivars have certain qualities or characteristics that allow the bacteria to colonize the plant, while the *Salmonella* Typhimurium is not able to do so in the cultivars examined in this study. Various other serovars of *Salmonella* in combination with other tomato cultivars should be tested to provide further evidence that a cultivar and/or serotype specificity for how well the bacterium can colonize the plant is present. Furthermore, additional routes of contamination should be investigated since studies have indicated the ability of *S. enterica* to be recovered from the fruit of tomato plants treated with contaminated irrigation water [34].

### 3.4. Persistence of E. coli O157:H7*-GFP* and Salmonella Typhimurium-GFP

**Figure 7:**
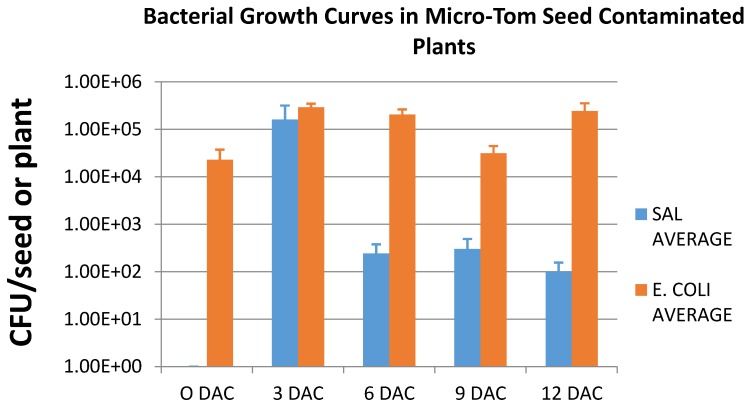
Change in microbial population (average CFU/plant) for Micro-Tom seeds that were contaminated with *Salmonella* Typhimurium-GFP (SAL Average) and *E. coli* O157:H7-GFP (E. COLI Average) and sampled 0, 3, 6, 9, and 12 days after contamination (DAC). Only colonies actively expressing GFP were included in the enumeration.

The data obtained for the growth curve of *E. coli* O157:H7-GFP and *Salmonella* Typhimurium-GFP that were actively expressing GFP following seed contamination of Micro-Tom plants also showed differences in how well the two bacteria were able to grow and persist on the plants (Figure 7). In the growth experiments, *E. coli* O157:H7-GFP was able to grow and persist better on young Micro-Tom plants in comparison to *Salmonella* Typhimurium-GFP. After the initial 30 min contamination, an average of 10^4^ CFU/seed could be enumerated from the *E. coli* O157:H7-GFP contaminated seeds. Following the same length of contamination, however, *Salmonella* Typhimurium-GFP could not be detected on or in the seeds (<100 CFU/seed). For the *E. coli* O157:H7-GFP treated plants, the average CFU/plant increased to 10^5^ after 3 days and this level was maintained throughout the 12 day sampling period with a decrease to an average of 10^4^ CFU/plant at 9 days following contamination. For the *Salmonella* Typhimurium-GFP, the average CFU/plant increased to 10^5^ after 3 days, however, this decreased to 10^2^ CFU/plant at 6 days following contamination and was maintained for the 12 day sampling period. These data suggest that there are differences between the bacteria that allow *E. coli* O157:H7-GFP to grow and persist on the tomato plants chosen for this study more effectively than *Salmonella* Typhimurium-GFP.

## 4. Conclusions

This study has shown that successful pathogen contamination of tomato fruit is dependent on or is an interaction of a specific pathogen, a specific tomato cultivar, and a specific route of contamination. These data indicate that prevention of contamination is critical and that the immediate plant environment should be closely monitored. Poor plant health or poor fruit bearing capabilities could provide a visible indication of contamination since most of the tomato cultivars in this study responded as such, but such an indication should not be used as a primary measure since some cultivars could appear healthy even when contaminated. Regardless, the ability of human pathogens to travel through the plant to the mature fruit, irrespective of route of contamination, is concerning. A further understanding of what bacterial species are able to colonize various tomato cultivars and what routes of contamination are important to produce contaminated fruit is important in developing mitigation strategies to prevent contamination, resulting in enhanced public health and economic well-being of the tomato industry.
